# Serum exosomes can restore cellular function *in vitro* and be used for diagnosis in dysferlinopathy: Erratum

**DOI:** 10.7150/thno.62181

**Published:** 2021-06-08

**Authors:** Xue Dong, Xianjun Gao, Yi Dai, Ning Ran, HaiFang Yin

**Affiliations:** 1 Department of Cell Biology, Tianjin Medical University, Qixiangtai Road, Heping District, Tianjin, 300070, China; 2 Department of Neurology, Peking Union Medical College Hospital, Chinese Academy of Medical Sciences, Shuaifuyuan Street, Dongcheng District, Beijing, 100730, China

The authors regret that an error was introduced in Figure 5E when the first author assembled the figure. In the original Figure 5E, the internal loading control of CD9 for detecting samples of P5, P10, P12, P13, P18 and P6, P8, P16, P19, P21 was repeatedly used instead of the right image corresponding to the samples used for this manuscript. The correct version is shown below. This image substitution would not affect any results presented in the originally-published version, nor the corresponding text description and the conclusion of the paper. The authors apologize for any inconvenience or misunderstanding that this error may have caused.

## Figures and Tables

**Figure 5:**
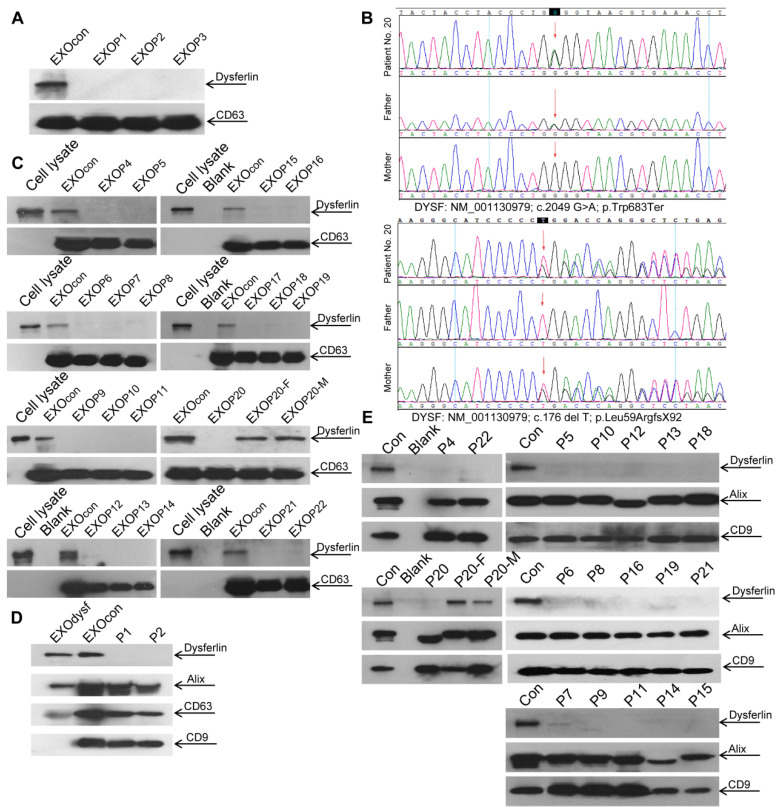
** Examination of dysferlin proteinin exosomes from normal controls' and dysferlinopathic patients' serum and urine**. (**A**) Western blot to examine levels of dysferlin protein in exosomes from normal controls' and dysferlinopathic patients' serum. 30 μg total protein from exosomes was loaded. Pn stands for the patient No. EXOcon refers to exosomes from normal controls' serum. (**B**) Representative gene sequencing results for dysferlinopathic patients (patient no. 20 and her parents). (**C**) Western blot to analyze the expression of exosomes derived from a spectrum of dysferlinopathic patients' serum. P20-F and P20-M mean patient no.20's father and mother. (**D**) Western blot to examine levels of dysferlin protein in exosomes from normal controls' and dysferlinopathic patients' urine. 50 μg total protein from exosomes was loaded. EXOcon refers to exosomes derived from normal human urine. (**E**) Western blot to analyze the expression of dysferlin in urine exosomes derived from a spectrum of dysferlinopathic patients.

